# Editorial

**Published:** 1872-08

**Authors:** 


### Editorial.

***Nostrum.***

A medicine, the ingredients of which are kept secret for the purpose of restricting the profits of sale to the inventor or proprietor. (Webster.)

Literally *our own;* applied to quack medicines retained for profit in the hands of the inventor or discoverer, or of his assignee (Brande.)

There is scarcely a druggist in this city who has not a private memorandum book filled with recipes belonging properly to the above category; and a file loaded with prescriptions for Dr. A.'s pills, Dr. B.'s cough syrup, Dr. C.'s cholera mixture, and so on, through the whole alphabet of names and catalogue of nostrums.

Many of these nostrum proprietors, too, earnestly, and apparently in good faith, decry the use, by their ignorant patients, of patent medicines, so-called, upon the principle that "two of a trade never agree." And yet, in what do their practices differ from those of Hembold, Radway & Co., except in being conducted upon a smaller scale with less profit?

The principle involved is the same in both cases. The physician, or, more properly speaking, the "medicine man," who directs a dozen patients indiscriminately to go to Blank's drug store and get some of "my mixture" or whatever else it may happen to be, without taking the trouble to make an especial diagnosis in each particular case (if he perchance be able to make one), is as much a quack as the veriest charlatan whose name disgraces the local columns of the daily papers.

Adapt the remedy to the disease, not the disease to the remedy, as in this procrustean practice. H.

### *Chicago Relief Fund.*

The Junior Editor of the JOURNAL, as Secretary of the late Medical Relief Committee, takes pleasure in acknowledging the receipt of one hundred and thirty-four dollars and fifty-one cents from physicians of New York, through Dr. S. T. Hubbard, Treasurer, and also of fifteen dollars from physicians of Lansingburgh, N. Y., through Dr. Geo. H. Hubbard.

As this money was received after the accounts of the Chicago Relief Committee had been finally audited and the committee discharged, the late secretary having no authority to disburse the funds, wrote to the donors offering to return the checks or to disburse the money at his own discretion by their authority. The latter alternative having been approved by the donors, and the necessary authority having been forwarded, the Junior Editor, with the co-operation of Dr. Ernst Schmidt, one of the members of the late committee, has disbursed one hundred dollars for the purposes and objects designated in the bequest.

There is now remaining in the hands of the Junior Editor the sum of forty-nine dollars and fifty-one cents, to be applied to the relief of any (of those designated by the donors) who may stand in need of it.

By to-day's mail we are informed by Dr. S. T. Hubbard, late treasurer of the New York Executive Committee, that a list of the names of the contributors to the Chicago Medical Relief Fund, in New York, will be forwarded. When this is received we shall take much pleasure in publishing it in the JOURNAL, as a permanent record, worthy to stand for all time, of the noble deeds of noble men. We would like to print it in letters of gold, or engrave it upon "perennial brass," if, in this, it could be more deeply impressed than it is, we are sure, in the grateful hearts of the medical men of Chicago, both of those who suffered and those who sympathized.

We subjoin the list of names above referred to:

Drs. Acheson, J.; Agnew, C. R.; Althoff, Herman; Allen, Charles M.; Angell, E. C.; Anderson, James; Assenheimer, August; Aub, Joseph.

Drs. Barker, Fordyce; Bahan, T. S.; Ball, A. Brayton; Balser, William; Banks, J. L.; Baetsendorf, H.; Beldon, E. B.; Bernachi, Charles; Beekman, John; Billington, C. E.; Bishop, John; Blakeman, W. N.; Blake; Blume, Samuel; Blumenthal, Mark; Bozeman, N.; Bowden, J. W.; Bradley, E.; Bopp, Ludwig; Brennan, J. W.; Budden, C. K.; Brown, J. L.; Burke, J.; Burrall, F. A.: Buchanan, Alexander; Budd, Charles A.

Drs. Castle, F. A.; Cash; Cash; Cash; Cash; Cash, F. C. F.; Cash; Cash; Cash, J. C. S.; Cash, J. J. D.; Cann, D. C.; Cash; Cash; Cash; Cash; Ceccanni, G.; Chamberlain, W. M.; Cheeseman, T. M.; Church, A. S.; Chaveau, J. F.; Clarke, P. J., Clark, A.; Conway, Wm. A.; Colby, J. L.; Cook, D.; Crampton, Henry C.; Curtis, E. & J.; Caswell, Hazard & Co., (Apoth.)

Drs. Dalton, J. C.; Davis, H. H.; Detmold, William; Delafield, Edward; Dessau, S. Henry; Derby, R. H.; Dew, J. H.; Dieffenbach, K. G. S.; Downs, Henry S.; Douglass, J. H.; Draper, Wm. C.; Du Bois, M. B.; Du Bois, A.; Du Bois, H. K.; Dunster, E. S.

Drs. Eager, Wm. B.; Emmett, Thomas Addis; Ellis, T. C.; Eno, H. C.

Drs. Fairbrother, C. M.; Ferber, Amandus; Feuchtwanger, L.; Finlay, Edward S.; Fisher, T. L.; Flint, Austin, Senior; Foster, Joel; Foscaro; Fowler, Edmund; Ford & Ridel; Frankel, J.; Frankenstein; Friend; Friend of Dr. J. Foster Jenkins; Frothingham, Wm.; Fuller, R. M.

Drs. Gardner, A. K.; Gay, H. S.; Geillahim, Wm.; Gabe, L. M.; Guleke, H.; Glasser, Joseph; Gomez, H.; Goldschmidt, L.; Griswold, Samuel; Gregory, H. H.; Gunn, A. N.

Drs. Hamilton, Frank; Hammond, W. A.; Harrison, A. J.; Hall, E.; Hadden, James; Hall, J. J.; Hahn, H.; Harrison, G. T.; Hart, Le Baron; Hall, Samuel; Hahn, S.; Herzog, Max; Herzberg, E.; Hirsch, S.; Hogan; Holgate, T. H.; Howard, Benj.; Hubbard, S. T.; Humphreys, George H.; Hue, Jude; Hunter, A. S.; Hunter, Wm. C.; House Staff, Strangers' Hospital; Hilton, H. R., U. S. A.

Infants' Hospital.

Drs. Janvrin, J. E.; Jacobi, A.; Jackson, W. H.; Johnson, A.; Judson, A. B.; Jay, A. S.; Johnson, W. H.; Janes, E. H.; Janeway, E. G.

Drs. Kammerer, J.; Keyes, E. L.; Kent; Kepler, A.; Keeney, B. M.; Kerrigan, J. A.

Drs. Kellogg, John M.; Kellogg, T. H.; Kissam, J. B.; Klapper; Knight, James; Knapp, H.; Kremer, Charles; Kost, H.; Krog, Carl; Koekler, August; Krackowizer, Ernst; Kessler, A.

Drs. Lang, Raphael; Langmain, G.; Lambert; Leale, C. A.; Learning, J. R.; Levings, N. B.; Little, J. L.; Loomis, A. L.; Loving, Edward; Lusk, Wm. T.

Drs. McClain, H.; McClelland, J.; McMillan, Charles H.; McNeily, R.; McQuestion, C. B.; McLarry, A. W.; Mattson, N.; Magie, David; Maxwell, E. R.; Mason, R. Q.; Maclay, A. W.; Medical Class, College of Physicians and Surgeons; Messenger, D.; Meyer, G. H.; Miller, Charles; Miller, D. B.; Miner, J. C.; Moses, M. J.; Moloney, A. A.; Morris, S. F.; Molin, Charles; Morrell, J. S.; Muller, R.; Mourraille, G.; Members Medical Journal Office.

Drs. Newman, R.; Neil, J.; Neftel, W. B.; Noeggerath, Emil; Nordeman, F.; Nott, J. C.; Noyes, H. D.

Drs. O'Dwyer, J.; O'Meagher, W.; O'Neill, D. E.; Obbarius, Herman; Orton, S. H.; Otis, F. R.

Drs. Parker, Willard; Parson, R. L.; Packard, C. W.; Peaslee, E. R.; Peters, J. C.; Piffard, H. G.; Plimer; Place, Nelson, Jr.; Post, A. C.; Pooler, H. A.; Purdy, A. E. M.; Purdy, A. S.; Pullin, E. R.

Drs. Raphael, B. J.; Railton, H. J.; Reynolds, James B.; Ripley, I. H.; Rogers, Stephen; Robinson, G. W.; Roof, S. W.; Russell, Charles P.; Robinson, J.

Drs. Sass, Luis F.; Sands, H. B.; Sayre, L. A.; Sabine, G. A.; Salvatore, Caro; Sewall, J. G.; Seguin, E. C.; Schwedler, E. F.; Sims, J. Marion; Simroch, Francis; Shrady, John F.; Shiff, Henry; Smith, O.G.; Smith, Governor M.; Smith, James O.; Smith, J. L.; Smith, A. H.; Stanley, C. G.; Sturgis, F. R.; Sundry, from Dr. Taylor; Stoddart, M.; Stackelberg, J.; Students Bellevue Hospital Medical College; Stein, S.; Steiger, Joseph; Stoddart, Alex. (layman); Sweeney, James; Swan, John O.; Satterlee; Sabine, Thomas F.; Stein, Charles.

Drs. Taylor, R. W.; Teats, S.; Teller, S.; Thebaud, J. S.; Thomas, T. G.; Thomson, B. S.; Thomson, Wm. H.; Toal, David D.; Tucker, C. N.; Tully, Marcus C.

Dr. Underhill, Alfred.

Drs. Van Antwerp, A.; Van Buren, Peter; Van Winkle, E. H.; Varley, C. D.; Vedder, Maus R.

Drs. Walton, L. P.; Watson, Wm. S.; Warner, J. W.; Ward, C. S.; Walton, C. J.; Warner, E. B.; Walser, Henry; Weir, R. F.; Weiner, J.; Weisman, F. H.; Wilson, J. H.; Williams, M. H.; Wheelock, G. G.; Wood, E. G.; Woolfarth, A.; Wood, J. R.; Wood, C. S.; W. S. D.; W. H. D.; Wuber, L.

Yorkville Medical Association.

Contribution from Naval Medical Officers, Brooklyn Navy Yard, through Dr. Geo. Peck, U. S. N.

Drs. Chase, C.; Henderson, A. A.; Jackson, S.; Keshneu, E.; Kidder, J. H.; Mackie, B. S.; Oberly, A. S.; Peck, George; Pilcher, L. S.; Schofield, W. K.; Smith, T. L.; White, C. S.; Willard, L. T.

Surgical and other instruments were also sent to Chicago through Dr. Hamilton, Chairman of the Executive Committee, viz. :

Mr. Stohlman sent several.

Dr. E. G. Rawson, a case of amputating instruments ; also a case of trephining instruments.

Messrs. Shephard & Dudley, a case of instruments to Woman's State Hospital.*

Dr. Eager, Gouley's instrument for stricture.*

Messrs. Otto & Reynders, sent an assortment of uterine instruments to Dr. A. B. Jackson, for use of the Woman's State Hospital.*

Otto & Reynders sent to the Christ Church Dispensary, instruments ; also sundries given by Mr. Plankter.*

Messrs. Darrow & Co. sent one electro-magnetic machine ; also one to Louis Drescher ; also a case of pocket instruments.†

Darrow & Co. sent one electro-magnetic machine ; one also presented by Mr. Louis Drescher.†

Darrow & Co. also contributed a case of pocket instruments.†

Dr. Frank Hamilton contributed instruments.

The sum total in money sent for the relief of medical men of Chicago through the Executive Committee, Dr. Frank Hamilton, Chairman, amounts to five thousand nine hundred and thirty-four dollars and fifty-one cents ($5,934.51.)

The committee reported at the time of its discharge the receipt of five thousand eight hundred dollars ($5,800) from the New York Executive Committee, through Dr. S. T. Hubbard, Treasurer, and Dr. Frank Hamilton, Chairman.

This amount, together with one hundred and thirty-four dollars and fifty-one cts. received from the same source by the late secretary of the committee since its discharge, makes a total aggregate of $5,934.51, forwarded by the New York Executive Committee and received by the Chicago Relief Committee. Of this amount, $34.51 still remain in the hands of the late Secretary of the Relief Committee.

In addition to this balance there has been received from

* These instruments were not received by the committee.

† Statement duplicated probably by clerical error.

Lansingburgh, N. Y., through Dr. George H. Hubbard, fifteen dollars.

Making an unexpended balance of forty-nine dollars and fifty-one cents, to be used for the benefit of any who may be in need, of those entitled to this aid. H.

***Medical Evidence.***

What now is more needed than anything else, is a treatise on Spinal Affections. There is not now a book, essay or tractate, on the subject, which is more than fragmentary. Erichsen's Treatise on Railway Spine, etc., is the merest twaddle. There is no work on the subject which rises above the scorn of the most pitiful attorney. Who will give us something worthy the profession? not as an advocate, not as a defender. Who will separate for us malingering, hysteria, and the functional disorders of *habit*, from *bona fide* organic lesions. As it is now, the medical evidence in such cases is a roaring farce, and a byword of reproach.

